# Deep learning neural network prediction of postoperative complications in patients undergoing laparoscopic right hemicolectomy with or without CME and CVL for colon cancer: insights from SICE (Società Italiana di Chirurgia Endoscopica) CoDIG data

**DOI:** 10.1007/s10151-025-03165-9

**Published:** 2025-06-11

**Authors:** G. Anania, P. Mascagni, M. Chiozza, G. Resta, A. Campagnaro, S. Pedon, G. Silecchia, D. Cuccurullo, C. Bergamini, G. Sica, V. Nicola, M. Alberti, M. Ortenzi, R. Reddavid, D. Azzolina

**Affiliations:** 1 Department of Medical Science, University of Ferrara, Ferrara, FE Italy; 2 Fondazione Policlinico Universitario Agostino Gemelli IRCCS, Rome, RM Italy; 3 Institute of Image-Guided Surgery, IHU-Strasbourg, Strasbourg, France; 4 Department of Scienze Medico Chirurgiche e Medicina Traslazionale, University of Roma S. Andrea University Hospital, Rome, RM Italy; 5 Division of Laparoscopic and Robotic Surgery Unit, A.O.R.N. dei Colli Monaldi Hospital, Naples, NA Italy; 6 Department of Emergency Surgery, University Hospital of Careggi, Florence, FI Italy; 7 Minimally Invasive Unit, Department of Surgical Science, University Tor Vergata, Rome, RM Italy; 8 Department of Surgical Science, Policlinico Tor Vergata - University Tor Vergata, Rome, RM Italy; 9 Department of Mathematics and Informatics, University of Ferrara, Ferrara, FE Italy; 10 Department of General Surgery, Università Politecnica delle Marche, Ancona, AN Italy; 11 Division of Surgical Oncology and Digestive Surgery, Department of Oncology, San Luigi University Hospital, University of Turin, Orbassano, Turin, TN Italy; 12 Biostatistics and Clinical Trial Methodology Unit, Clinical Research Center DEMeTra, Department of Translational Medicine, University of Naples Federico II, Naples, Italy

**Keywords:** Postoperative complications, Colon cancer surgery, Deep learning neural networks, Predictive modeling

## Abstract

**Background:**

Postoperative complications in colorectal surgery can significantly impact patient outcomes and healthcare costs. Accurate prediction of these complications enables targeted perioperative management, improving patient safety and optimizing resource allocation. This study evaluates the application of machine learning (ML) models, particularly deep learning neural networks (DLNN), in predicting postoperative complications following laparoscopic right hemicolectomy for colon cancer.

**Methods:**

Data were drawn from the CoDIG (ColonDx Italian Group) multicenter database, which includes information on patients undergoing laparoscopic right hemicolectomy with complete mesocolic excision (CME) and central vascular ligation (CVL). The dataset included demographic, clinical, and surgical factors as predictors. Models such as decision trees (DT), random forest (RF), and DLNN were trained, with DLNN evaluated using cross-validation metrics. To address class imbalance, the synthetic minority over-sampling technique (SMOTE) was employed. The primary outcome was the prediction of postoperative complications within 1 month of surgery.

**Results:**

The DLNN model outperformed other models, achieving an accuracy of 0.86, precision of 0.84, recall of 0.90, and an F1 score of 0.87. Relevant predictors identified included intraoperative minimal bleeding, blood transfusion, and adherence to the fast-track recovery protocol. The absence of intraoperative bleeding, intracorporeal anastomosis, and fast-track protocol adherence were associated with a reduced risk of complications.

**Conclusion:**

The DLNN model demonstrated superior predictive performance for postoperative complications compared to other ML models. The findings highlight the potential of integrating ML models into clinical practice to identify high-risk patients and enable tailored perioperative care. Future research should focus on external validation and implementation of these models in diverse clinical settings to further optimize surgical outcomes.

## Introduction

Complications after surgery have a serious impact on many aspects of surgical care at several levels beginning from a significant disruption of patient recovery to an increased burden on healthcare costs [1–3]. The possibility to accurately predict the probability of occurrence could be used as a tool in prognosis prediction, tailored resource allocation, and perioperative decision-making [4]. This implies an impact on all surgical phases, from preoperative patient selection to intraoperative planning and postoperative management [5].

Intraoperative planning involves the choice of the potential best surgical technique, and, in times where innovation plays an important role in surgery, the availability of several techniques might have raised the challenge of this choice [6].

In recent years, complete mesocolic excision (CME) has been proposed to potentially improve oncological outcomes, particularly in laparoscopic right hemicolectomy [7]. The Italian healthcare system’s commitment to refining these techniques is evident through the CoDIG project. The CoDIG 1 and CoDIG 2 studies, involving extensive multicenter data, have provided insights into the surgical management of right hemicolectomy for colon cancer and highlighted the variability in surgical techniques and outcomes across different medical centers in Italy [8, 9]. The CME and central vascular ligation (CVL) are technically demanding procedures that require a high level of surgical skill and precision. They involve meticulous dissection along embryological planes and the ligation of central vascular structures to ensure complete removal of the cancerous tissue and associated lymph nodes​. This complexity could increase the risk of intraoperative and postoperative complications such as bleeding, anastomotic leaks, and infections [10].

Some studies have suggested potential oncological benefits of CME and CVL [11]. However, some evidence, such as the RELARC trial, did not demonstrate a significant benefit in disease-free survival (DFS) outcome for CME compared to standard D2 dissection in right-sided colon cancer [12].

As a result of the complexity and extended duration of CME and CVL surgeries, the surgical procedure requires healthcare resources, including specialized surgical teams, advanced equipment, and extended operating room times. Predicting postoperative complications helps optimize the use of these resources by anticipating the needs for postoperative care, including intensive care unit (ICU) admissions, specialized nursing care, and extended hospital stays. This optimization can lead to more efficient allocation of hospital resources, reducing costs and improving patient outcomes [13, 14].

In this general framework, the application of machine learning (ML) models in medical research has shown promise in improving predictive accuracy by handling complex datasets and uncovering non-linear patterns that traditional statistical methods may overlook [5]. Patients undergoing CME and CVL often present with varying levels of comorbidities, tumor stages, and overall health statuses [15]. In this clinical context, the ML models can analyze a wide array of patient-specific factors to predict the risk of complications, enabling personalized preoperative and postoperative care plans. This individualized approach may optimize the possibility that high-risk patients receive the necessary precautions and interventions, thereby improving their outcomes and reducing the incidence of adverse events [16]. Moreover, the data collected and analyzed through ML models could provide useful findings into the factors contributing to postoperative complications in CME and CVL [4]. Healthcare providers can refine surgical techniques, enhance training programs, and develop evidence-based guidelines to minimize risks by identifying patterns and trends via ML tools [17]. In this general framework, the application of ML in surgical outcome prediction, particularly in estimating the postoperative length of stay (LoS), has been shown in the literature promising results. Previous research utilizing the CoDIG database, for instance, has demonstrated that ML models can effectively predict LoS after laparoscopic right hemicolectomy for colon cancer, underscoring the potential for tailored patient care and optimized hospital resource allocation [13].

Concerning the postoperative complications, there are already existing tools for perioperative predictive analytic decision-support; however, tools are hindered by suboptimal performance, time constraints imposed by manual data entry requirements, and lack of intraoperative data and clinical workflow integration [18]. Despite its potential benefits, the application of ML to predict postoperative complications in laparoscopic surgery for colon cancer is still underexplored in the literature.

This study seeks to address this gap by proposing an ML-based tool designed to assess patient-specific risks of complications for those undergoing CME and CVL surgical procedures.

## Materials and methods

### Data

The overall pooled multicenter database CoDIG database has been considered for training the model [8, 9, 19]. The database includes information for 2013 patients undergoing laparoscopic right hemicolectomy with or without CME and CVL for colon cancer.

The overall dataset considered is the pooling of the data provided by CoDIG 1 and CoDIG 2 projects both endorsed by the Italian Society of Endoscopic Surgery and New Technologies (SICE). The CoDIG 1 study is a large, multicenter study aimed at evaluating the surgical outcomes associated with two different techniques of ileo-colic anastomosis (intracorporeal [ICA] and extracorporeal [ECA]) during laparoscopic right hemicolectomy. This prospective cohort study involved 85 surgical units across Italy, contributing data on 1225 patients who underwent elective laparoscopic or robotic right hemicolectomy between March 2018 and September 2018 [8]. The CoDIG 2 study is an observational, multicenter database composed of 788 patients, a national study involving Italian surgical wards specializing in colorectal surgery. It aimed to assess the practices of Italian surgeons regarding the extent of lymphadenectomy performed during right hemicolectomy for colon cancer. This study sought to understand the current surgical approaches and any evolving trends compared to the previous CoDIG 1 study [9, 19].

#### Outcome and variables

The primary outcome of the ML tool is the prediction of complications occurring within 1 month for patients undergoing right hemicolectomy with CME and CVL surgical procedures.

The predictors considered for training the model are selected according to the clinical judgment and include demographic, clinical, and surgical factors. Gender is categorized as female or male, while age is treated as a continuous variable. Comorbidities are classified as none or one or more, and the American Society of Anesthesiologists (ASA) score ranges from I to IV. The body mass index (BMI) is grouped into the following categories: < 18, 18–24, 25–30, and > 30. Pathology is designated as either benign or malignant, with previous surgery specified as none or yes. Tumor staging includes T stage (T1, T2, T3, T4) and N stage (N+, N0, N1), alongside M stage, recorded as either M+ or M0. The number of lymph nodes removed and surgery duration, categorized as > 270 min, 181–270 min, or 90–180 min, are also analyzed. Blood management variables include transfusion (no, yes) and intraoperative minimal bleeding (no, yes). Surgical technique is further specified with variables such as anastomosis (extracorporeal, intracorporeal), drainage (no, yes), conversion (no, yes), and fast-track protocol (no, yes). Finally, the right hemicolectomy type is classified as laparoscopic, robotic, or video-assisted.

### Algorithm development

#### Data preprocessing

Categorical variables were converted into numerical formats to facilitate their use in models. Binary categorical variables, such as gender, were encoded converting categories into binary values (0 or 1). For non-binary categorical variables with natural order, such as tumor stages (T), ordinal encoding was employed, assigning unique integers based on the severity of each category.

#### Class imbalance

The dataset exhibited a class imbalance, with 85% of cases showing no complications and 15% showing complications. To balance the dataset, the synthetic minority over-sampling technique (SMOTE) was applied.

#### Model training and validation

Several models were trained, including decision trees (DT), random forest (RF) ensemble models, and deep learning neural networks (DLNN).

- The *DT* is a model that splits the data into branches based on feature values, leading to a tree-like structure of decisions and their possible outcomes. It is intuitive and easy to interpret [20].
- The *ensemble models* combine multiple decision trees to improve prediction accuracy and robustness. Techniques such as *RF* fall into this category, leveraging the strengths of multiple models to enhance performance [21].
- The *DLNN* is a larger model consisting of multiple layers of interconnected neurons that can capture complex patterns in the data. The DLNN is defined to handle binary classification problems, where the objective is to predict the presence or absence of postoperative complications based on several input features. The model’s architecture includes three hidden layers with ReLU activation functions, L2 regularization to mitigate overfitting, and batch normalization to stabilize and accelerate the training process. The output layer uses a sigmoid activation function to generate a probability score for the binary classification task. The model is compiled with the RMSprop optimizer and binary cross-entropy loss function, chosen for their effectiveness in binary classification. Early stopping is implemented to halt training when the validation loss stops improving, thereby mitigating overfitting and ensuring the model retains the best weights observed during training [22].

The fivefold cross-validation was implemented to evaluate the model’s performance. The dataset was divided into five subsets (folds). The model was trained on four folds and validated on the remaining fold. This process was repeated five times, with each fold being used as the validation set once.

The early stopping procedure was employed to prevent overfitting by monitoring the validation loss and stopping training when the loss did not improve for 10 consecutive epochs. The best model during each fold was saved and based on the lowest validation loss to ensure optimal model parameters were retained.

### Statistical analysis

#### Descriptive statistics

Descriptive statistics of the data were presented using the median and interquartile range for quantitative variables, and absolute and relative frequencies for qualitative variables. To analyze differences between groups, the Wilcoxon test was applied for numerical features, while the chi-square test or, when appropriate, the Fisher exact test was used for categorical features. The univariable logistic regression model odds ratios with 95% confidence intervals have been also reported.

#### ML model performance

The performance of the models was assessed using several metrics in cross-validation, including accuracy, precision, recall, and F1 score assessed in cross-validation and on the training sample.

- *Accuracy* measures the overall correctness of the model. It is the proportion of true results (both true positives and true negatives) out of the total number of predictions made.
- *Precision* focuses on the accuracy of positive predictions. It is the ratio of true positives (correctly predicted positive outcomes) to all positive predictions (true positives and false positives).
- *Recall* (also called sensitivity) measures the ability of the model to identify all positive instances correctly. It is the ratio of true positives to all actual positives (true positives and false negatives).
- *F1 Score* is the harmonic mean of precision and recall, combining them into a single metric. It is useful when there is an uneven class distribution and when we seek a balance between precision and recall. The F1 score ranges from 0 to 1, with 1 being the best possible score, indicating high precision and recall.

Performance metrics before (first attempt) and after (second attempt) implementing SMOTE and cross-validation techniques are reported. A receiver operating characteristic (ROC) plot was also provided on the training sample.

#### Variable importance

To assess the relative influence of each predictor variable in our model, we employed permutation importance, a commonly used method for evaluating feature importance in machine learning models. This technique involves shuffling the values of each feature individually and measuring the decrease in model performance, typically quantified by area under the ROC curve (AUC). The larger the decrease in model performance, the more important the feature is deemed to be. The results were visualized in a bar chart, ranking the features by their importance score, which provided insights into the key drivers of postoperative complications in our study cohort.

The analyses have been performed in Python 9.3 [23] with TensorFlow package and R 3.4.2 [24].

## Results

An overall study sample of 2013 subjects has been considered for training the model. Table 1 reports the overall characteristics of the study population considered for training the ML. Multiple preexistent comorbidities (OR 1.23; 95% CI 1.02–1.48), T4 infiltration (OR of 1.94 95% CI 1.32–2.87), and intraoperative blood transfusion (OR 1.85; 95% CI 1.52–2.25) were identified as positive risk factors for postoperative complications.

**Table 1:** Descriptive statistics of the patient’s characteristics used for training the ML models

Variable	Overall *N* = 2013	No complications *N* = 1355	Complications *N* = 658	OR [95% CI]	*P*
Gender					0.469
Female	964 (47.9%)	657 (48.5%)	307 (46.7%)	Ref	
Male	1049 (52.1%)	698 (51.5%)	351 (53.3%)	1.08 [0.89; 1.30]	
Age	74.0 [66.0; 80.0]	74.0 [65.5; 80.0]	74.0 [66.0; 81.0]	1.00 [1.00; 1.01]	0.254
Comorbidities					0.038
None	1187 (59.0%)	821 (60.6%)	366 (55.6%)	Ref	
One or more	826 (41.0%)	534 (39.4%)	292 (44.4%)	1.23 [1.02; 1.48]	
ASA score					0.369
I	120 (5.96%)	75 (5.54%)	45 (6.84%)	Ref	
II	966 (48.0%)	663 (48.9%)	303 (46.0%)	0.76 [0.51; 1.14]	
III	840 (41.7%)	563 (41.5%)	277 (42.1%)	0.82 [0.55; 1.23]	
IV	87 (4.32%)	54 (3.99%)	33 (5.02%)	1.02 [0.57; 1.80]	
BMI					0.803
< 18	54 (2.68%)	38 (2.80%)	16 (2.43%)	Ref	
18–24	936 (46.5%)	632 (46.6%)	304 (46.2%)	1.14 [0.63; 2.13]	
25–30	758 (37.7%)	513 (37.9%)	245 (37.2%)	1.13 [0.63; 2.12]	
> 30	265 (13.2%)	172 (12.7%)	93 (14.1%)	1.28 [0.68; 2.48]	
Pathology					0.802
Benign	196 (9.74%)	134 (9.89%)	62 (9.42%)	Ref	
Malignant	1817 (90.3%)	1221 (90.1%)	596 (90.6%)	1.05 [0.77; 1.45]	
Previous surgery					0.319
None	1077 (53.5%)	714 (52.7%)	363 (55.2%)	Ref	
Yes	936 (46.5%)	641 (47.3%)	295 (44.8%)	0.91 [0.75; 1.09]	
T stage					< 0.001
T1	237 (14.0%)	169 (15.0%)	68 (11.9%)	Ref	
T2	386 (22.7%)	279 (24.8%)	107 (18.7%)	0.95 [0.67; 1.37]	
T3	853 (50.3%)	554 (49.2%)	299 (52.4%)	1.34 [0.98; 1.84]	
T4	221 (13.0%)	124 (11.0%)	97 (17.0%)	1.94 [1.32; 2.87]	
N stage					0.009
N+	212 (12.5%)	124 (11.0%)	88 (15.3%)	Ref	
N0	1084 (63.7%)	744 (66.0%)	340 (59.2%)	0.64 [0.48; 0.87]	
N1	406 (23.9%)	260 (23.0%)	146 (25.4%)	0.79 [0.56; 1.11]	
M stage					0.273
M+	99 (5.86%)	60 (5.37%)	39 (6.83%)	Ref	
M0	1589 (94.1%)	1057 (94.6%)	532 (93.2%)	0.77 [0.51; 1.18]	
Lymph nodes removed	21.0 [16.0; 28.0]	22.0 [16.0; 28.0]	20.0 [15.0; 27.0]	0.99 [0.98; 1.00]	0.074
Surgery duration					0.501
> 270 min	158 (7.85%)	108 (7.97%)	50 (7.60%)	Ref	
181–270 min	693 (34.4%)	477 (35.2%)	216 (32.8%)	0.98 [0.68; 1.43]	
90–180 min	1162 (57.7%)	770 (56.8%)	392 (59.6%)	1.10 [0.77; 1.58]	
Transfusion					< 0.001
No	839 (41.7%)	629 (46.4%)	210 (31.9%)	Ref	
Yes	1174 (58.3%)	726 (53.6%)	448 (68.1%)	1.85 [1.52; 2.25]	
Intraoperative minimal bleeding					< 0.001
No	1229 (61.1%)	753 (55.6%)	476 (72.3%)	Ref	
Yes	784 (38.9%)	602 (44.4%)	182 (27.7%)	0.48 [0.39; 0.58]	
Anastomosis					< 0.001
Extracorporeal	573 (28.5%)	327 (24.1%)	246 (37.4%)	Ref	
Intracorporeal	1440 (71.5%)	1028 (75.9%)	412 (62.6%)	0.53 [0.44; 0.65]	
Drainage					0.602
No	752 (37.4%)	512 (37.8%)	240 (36.5%)	Ref	
Yes	1261 (62.6%)	843 (62.2%)	418 (63.5%)	1.06 [0.87; 1.28]	
Conversion					0.058
No	1888 (93.8%)	1281 (94.5%)	607 (92.2%)	Ref	
Yes	125 (6.21%)	74 (5.46%)	51 (7.75%)	1.46 [1.00; 2.10]	
Fast-track protocol					< 0.001
No	817 (40.6%)	481 (35.5%)	336 (51.1%)	Ref	
Yes	1196 (59.4%)	874 (64.5%)	322 (48.9%)	0.53 [0.44; 0.64]	
Right hemicolectomy type					0.812
Laparoscopic	1736 (86.2%)	1164 (85.9%)	572 (86.9%)	Ref	
Robotic	185 (9.19%)	127 (9.37%)	58 (8.81%)	0.93 [0.67; 1.28]	
Video-assisted	92 (4.57%)	64 (4.72%)	28 (4.26%)	0.89 [0.56; 1.40]	

Patients with no intraoperative blood loss (OR 0.48 [95% CI 0.39–0.58]), intracorporeal anastomosis (OR 0.53 [95% CI 0.44–0.65]), and managed following a fast-track recovery protocol (OR 0.53 [95% CI 0.44–0.64]) had a significantly lower risk of complications.

The results evidenced the superior performance of the DLNN model compared to RF and DT models across several key metrics, as assessed through cross-validation (Table 2, panel A). In terms of accuracy, the DLNN model achieved the highest score at 0.86. This performance is better than the RF model’s accuracy of 0.74 and the DT model’s accuracy of 0.65, underscoring the DLNN’s superior predictive power. The precision of the DLNN model was 0.84, indicating that 84% of the predicted complications were actual complications. This metric was higher than the precision of the RF model, which was 0.78, and much higher than that of the DT model, which stood at 0.64. This suggests that the DLNN model produces fewer false positives compared to the other models. The recall was particularly high for the DLNN model at 0.90. This indicates that the DLNN was very effective at identifying patients who would experience complications, with a significantly lower rate of false negatives. In comparison, the RF and DT models both achieved a recall of 0.69. The F1 score was 0.87 for the DLNN model. The F1 score for the DLNN was higher than the RF model’s score of 0.73 and the DT model’s score of 0.67.

**Table 2:**
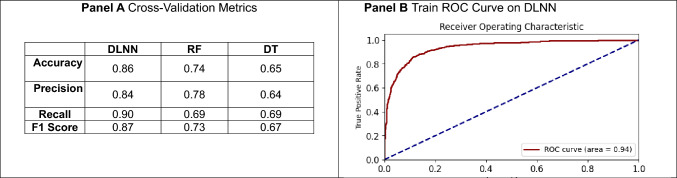
Performance metrics

The ROC plot (Table 2, panel B) represents the ability of the DLNN model to predict the occurrence of complications; the AUC was 0.94.

**Fig. 1:**
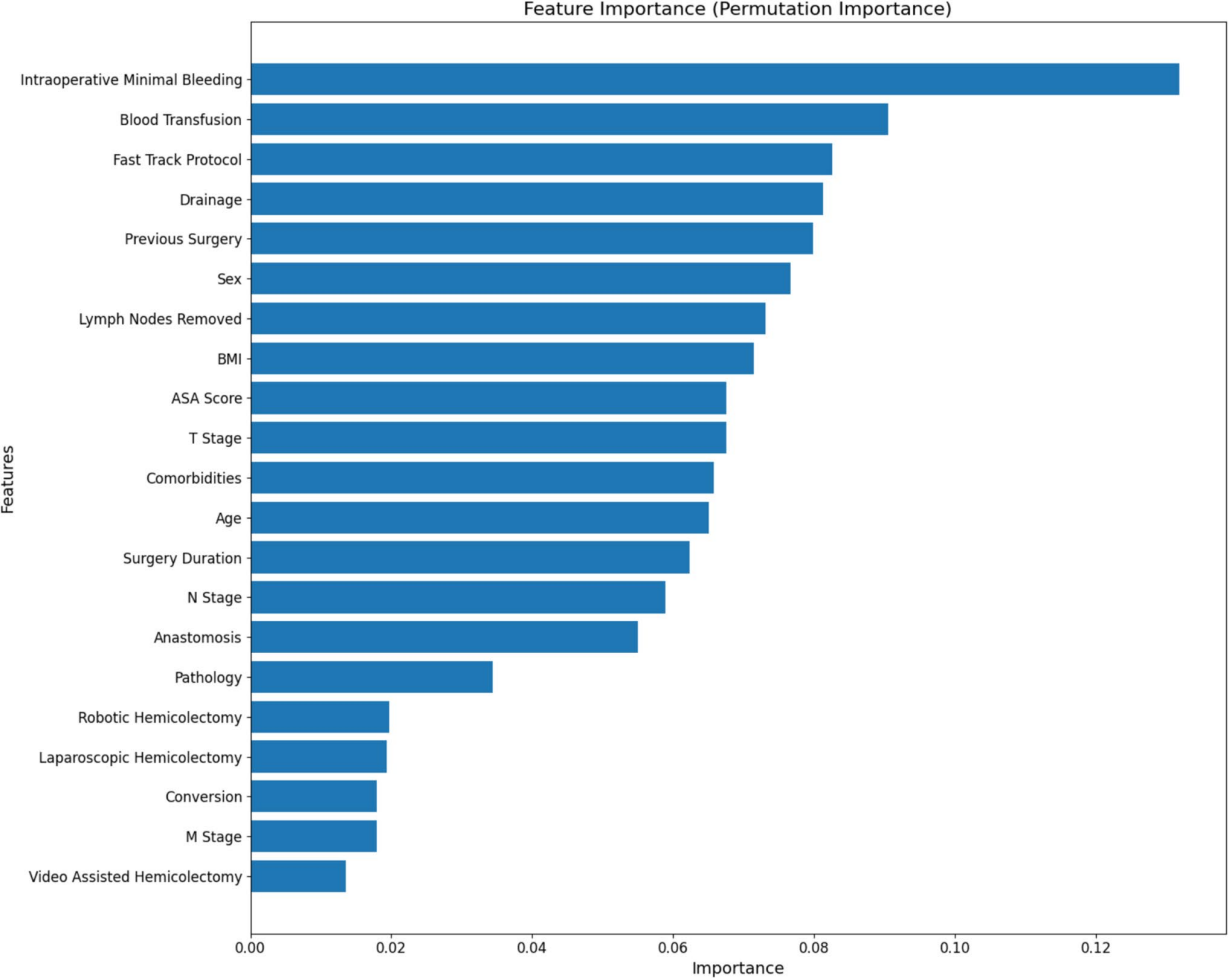
Variable importance plot

The plot in Fig. 1 illustrates the feature importance of various factors in predicting postoperative complications, as determined by permutation importance. The most influential feature is intraoperative minimal bleeding, which stands out with the highest importance score. This is followed by blood transfusion and fast-track protocol, both of which also show a significant impact on the prediction model.

Performance metrics before (first attempt) and after (second attempt) implementing SMOTE and cross-validation techniques are reported in Table 3.

**Table 3:** Performance metrics of DNN model before (first attempt) and after (second attempt) implementing SMOTE and cross-validation techniques

Metric	First attempt	Second attempt
Accuracy	0.73	0.86
Precision (complications)	0.46	0.84

## Discussion

The study demonstrated that advanced ML techniques, particularly DLNN, improve the predictive accuracy for postoperative complications. The DLNN model achieved the highest accuracy, surpassing the RF, and DT with cross-validation.

Previous studies have established that for many clinical prediction tasks, deep neural networks outperform other methods, such as logistic regression classifiers [25, 26]. The singularity of the study stands on the application of these models on a novel and debated technique and on the heterogeneous nature of the dataset.

Several variables were identified as impacting factors on the risk of postoperative complications. Comorbidities played an important role, with patients having one or more comorbidities showing a higher risk of complications [27]. Tumor staging was another critical factor; patients with T4-stage tumors had nearly double the risk of complications compared to those with T1-stage tumors [28]. Additionally, the need for intraoperative blood transfusions was associated with a higher risk of complications [29]. Conversely, the absence of intraoperative blood loss and the use of intracorporeal anastomosis were associated with significantly lower risks of complications [30].

Instead, in training the algorithm, the feature importance used for training the ML model evidenced that the most influential variable identified is the intraoperative minimal bleeding, which has the highest importance score. This underscores the importance of blood management during surgery. Minimizing intraoperative bleeding is crucial, suggesting that meticulous surgical techniques and effective hemostasis are essential in reducing the risk of complications. Surgeons and operative teams should prioritize strategies that minimize blood loss to improve patient outcomes [31, 32].

Blood transfusion is the second most important feature, indicating that patients requiring transfusions are at a higher risk of developing complications within 1 month. This finding stresses the need for careful intraoperative monitoring and decision-making regarding transfusions. Reducing the need for transfusions through preoperative optimization of patient hemoglobin levels and using blood conservation techniques can be beneficial [33]. For patients who do require transfusions, enhanced postoperative monitoring and supportive care are critical to mitigate the associated risks [34].

The fast-track protocol also shows a significant impact on the prediction model. The fast track protocol—emphasizing early mobilization, optimized pain control, and minimal perioperative fasting—highlights its effectiveness in improving and reducing complications [35]. This is consistent with enhanced recovery after surgery (ERAS) protocol research [35], which regularly demonstrates that these practices lead to quicker recovery, fewer complications, and higher patient satisfaction, thereby benefiting both clinical outcomes and operational efficiency [36]. Implementing fast-track protocols involves coordinated efforts to follow evidence-based perioperative practices, such as optimal pain management, early mobilization, and nutritional support. These protocols should be adopted widely, ensuring that all elements are rigorously applied to maximize their benefits [36]. Despite this evidence, ERAS protocols have seen limited adoption in Italy, with organizational challenges in modifying established care pathways cited as a major barrier. Implementing ERAS requires substantial resources and places high demands on the multidisciplinary team. Additionally, recent reviews highlight various perioperative management programs [37], each differing considerably in components and adherence levels [38].

Our findings align with those of recent studies. For example, the literature identified relevant factors influencing postoperative LoS in right hemicolectomy patients, the use of fast-track protocols, anastomosis type, and drainage [13]. Our model extends these insights by incorporating additional intraoperative and demographic variables, which may improve predictive accuracy and offer a more comprehensive tool for clinical decision-making.

From a practical standpoint, these insights have implications for the operating room: surgeons and operative teams must adopt a multidisciplinary approach, focusing on the top predictors such as minimizing intraoperative bleeding, carefully managing blood transfusions, and rigorously implementing fast-track protocols. This approach can lead to more personalized patient care and optimized resource utilization, thereby improving surgical outcomes.

From the computational standpoint, the implementation of cross-validation and SMOTE [39] was useful in improving the model’s performance. These techniques addressed class imbalances and ensured that the model was generalizable. The comparison of model performance before and after these improvements revealed substantial gains, with accuracy and precision for both complications and non-complications rising significantly.

The integration of ML models into clinical practice has several implications. By accurately predicting which patients are at higher risk of postoperative complications, healthcare providers can tailor perioperative care to individual patient needs, potentially reducing the incidence of adverse events, shortening hospital stays, and improving overall patient outcomes [13]. The continuous learning capability of ML models also means that their predictive accuracy can improve over time as more data becomes available [40].

Clinically, high-risk patients can be identified perioperatively, allowing for targeted interventions such as more rigorous monitoring, prophylactic treatments, or surgical adjustment [13]. These findings confirm what has been previously stated, with the novelty of the possibility of applying in a more efficient way supported by an ML tool.

To be safely implemented into the clinical practice, this tool has to demonstrate a high level of accuracy. Future research should focus on expanding the dataset and externally validating the algorithm with more diverse patient populations and additional variables. The practical implementation of these models in clinical settings, including the development of user-friendly interfaces for healthcare providers, should also be explored to maximize their clinical impact.

## Conclusions

This study demonstrated that DLNN improves the predictive accuracy for postoperative complications in patients undergoing laparoscopic right hemicolectomy for colon cancer with or without CME and CVL. The most influential predictors identified were intraoperative minimal bleeding, blood transfusions during surgery, and the implementation of fast-track recovery protocols. The application of models to predict postoperative complications in patients undergoing CME and CVL for colon cancer has shown promising results. The findings from this study underscore the potential of ML to tailor clinical decision-making and patient care by providing a more accurate risk prediction tool.

The findings highlight the potential of integrating DLNN models into clinical practice to improve surgical outcomes and overall patient care. Future research should focus on expanding datasets and further validating these models to maximize their clinical impact.

## Data Availability

The data that support the findings of this study are not openly available due to confidentiality reasons and are available from the corresponding author upon reasonable request.
